# Supplementary material for: Synergistic Effects of Sanglifehrin-Based Cyclophilin Inhibitor NV651 with Cisplatin in Hepatocellular Carcinoma
Source: Cancers (Basel). 2022 Sep 20;14(19):4553. doi: 10.3390/cancers14194553 (PMC9559492; doi:10.3390/cancers14194553)
Supplement: Supplementary file 1 [file cancers-14-04553-s001.zip › cancers-1853872-supplementary.pdf]

Table S1. AUC, tissue of origin and availability of transcriptomics data for 50 cell lines.

Cell Line	Tissue	HBV	Transcriptome Availability	NV651 IC50 (nM)	NV651 AUCs	NV651 Sensitivity
BXPC3	Pancreas		yes	59.99	1.98	Insensitive
NOZ	Liver	-	yes	51.84	2.10	Insensitive
SW1990	Pancreas		yes	34.41	2.26	Insensitive
HCT116	Colorectum		yes	71.80	2.36	Insensitive
RKO	Colorectum		yes	79.79	2.55	Insensitive
OZ	Liver	-	yes	83.79	2.55	Insensitive
CAPAN1	Pancreas		yes	157.49	2.55	Insensitive
HT29	Colorectum		yes	101.19	2.58	Insensitive
LOVO	Colorectum		yes	163.75	2.59	Insensitive
HUCCT1	Liver	-	yes	194.01	2.59	Insensitive
SNU739	Liver	+	yes	349.94	2.72	Insensitive
JHH4	Liver	-	yes	485.64	2.77	Insensitive
SW480	Colorectum		yes	164.66	2.79	Insensitive
MIAPACA2	Pancreas		yes	210.22	2.82	Insensitive
HEPG2	Liver	-	yes	437.43	2.86	Insensitive
SKHEP1	Liver	-	yes	1266.92	2.88	Intermediate
JHH7	Liver	+	yes	595.20	2.93	Intermediate
DLD1	Colorectum		no	657.24	2.93	Intermediate
T84	Colorectum		yes	478.95	3.00	Intermediate
CFPAC1	Pancreas		yes	1334.05	3.03	Intermediate
JHH6	Liver	-	yes	3252.59	3.07	Intermediate
HUH6	Liver	-	yes	NA	3.08	Intermediate
PLC/PRF/5	Liver	+	yes	NA	3.13	Intermediate
OCUG1	Liver	-	yes	NA	3.13	Intermediate
HLE	Liver	-	yes	2932.07	3.13	Intermediate
SNU398	Liver	+	yes	NA	3.16	Intermediate
SNU475	Liver	+	yes	NA	3.22	Intermediate
LS411N	Colorectum		yes	NA	3.22	Intermediate
HCT8	Colorectum		no	1971.11	3.27	Intermediate
KP4	Pancreas		yes	NA	3.28	Intermediate
PL45	Pancreas		no	NA	3.28	Intermediate
HUH7	Liver	-	yes	NA	3.29	Intermediate
HLF	Liver	-	yes	1379.77	3.29	Intermediate
HCT15	Colorectum		yes	2159.44	3.33	Intermediate
PANC1	Pancreas		yes	NA	3.34	Intermediate
HCCLM3	Liver	+	yes	NA	3.37	Intermediate
HCCC9810	Liver	-	yes	NA	3.37	Intermediate
LI7	Liver	-	yes	3599.92	3.39	Sensitive
SNU387	Liver	+	yes	NA	3.51	Sensitive
SNU423	Liver	+	yes	NA	3.52	Sensitive
HUH1	Liver	-	yes	285920.55	3.52	Sensitive
MHCC97H	Liver	+	yes	NA	3.55	Sensitive
ASPC1	Pancreas		yes	19507.22	3.69	Sensitive
SNU354	Liver	+	yes	135657.60	3.70	Sensitive
SNU761	Liver	+	yes	NA	3.82	Sensitive
JHH5	Liver	-	yes	10235.99	3.87	Sensitive
SNU368	Liver	+	yes	NA	3.91	Sensitive

JHH1	Liver	-	yes	30722.03	4.04	Sensitive
SNU449	Liver	+	yes	31827.04	4.09	Sensitive
HEP3B217	Liver	+	yes	NA	4.17	Sensitive

*Cell lines are arranged in ascending order in relation to Area Under the curve (AUC). HBV DNA detected and/or hepatitis B surface antigen (HBsAg) expression in liver derived-cell lines are indicated as HBV positive. The AUCs were normalized using z-score method: z-score > 0.5 (insensitive) and z-score < -0.5 (sensitive), which corresponds to the original AUC values of 3.39 and 2.86.

Table S2. Primers for quantitative PCR for human.

BRIP1-F qPCR	TGTCTGTTGATATTCTGAGTCGAT
BRIP1-R qPCR	CCCTAAATGTAAATGAGTAAGAGCC
FANCA-F qPCR	CAGAACCCAACTCTGCTGAGGA
FANCA-R qPCR	ATCACTGCCACCTGTGCCGATA
FANCB-F qPCR	GGACTCCTTGAACTCAGACTGC
FANCB-R qPCR	AGAGGTGGAACCACCAGGTAAC
FANCL-F qPCR	GGAGTGCAACAGCACGCAGAAT
FANCL-R qPCR	CTGCTCAGCTTAATTCCCAGGG
REV1-F qPCR	GAAATCCACCGAAGAGGAGCAC
REV1-R qPCR	ATCAGGACTGGTCGGCAGATGT
POLA2-F qPCR	TTCGTGAAGGATGTCCTCGG
POLA2-R qPCR	TGACGACCTGCACAGCAAT
PRIM1-F qPCR	ACTGTTTTCCACGGCTGGAT
PRIM1-R qPCR	AATGGCATCCAATTCACGGC
RFC3-F qPCR	CCTGAGACAGATTGGGAGGTGT
RFC3-R qPCR	AGCTCATACAGCCTTCCACGAAC
RFC4-F qPCR	GGCAGCTTTAAGACGTACCATGG
RFC4-R qPCR	TCTGACAGAGGCTTGAAGCGGA
ATM-F qPCR	TGCGTGGCTAACGGAGAAAA
ATM-R qPCR	CACTGTCACTGCACTCGGAA
E2F3-F qPCR	AGCGGTCATCAGTACCTCTCAG
E2F3-R qPCR	TGGTGAGCAGACCAAGAGACGT
SMAD2-F q	GGGTTTTGAAGCCGTCTATCAGC
SMAD2-R	CCAACCACTGTAGAGGTCCATTC
GADD45A-F	AATTCTCGGCTGGAGAGCAG
GADD45A-R	TACACCCCGACAGTGATCGT
ORC2-F	GAGGAGAGAAGAGCCAGCAAATC
ORC2-R	GACTCTGCTTTGCATGATCCCAC
ORC6-F	GAGAAGATTGGACAGCAGGTCG
ORC6-R	GGTTTATGTGGCATCTCCTCTAC
BRCA1-F	CTGAAGACTGCTCAGGGCTATC
BRCA1-R	AGGGTAGCTGTTAGAAGGCTGG
DNA2-F	GATTTCTGGCACCAGCATAGCC
DNA2-R	ACACCTCATGGAGAACCGTACC
GAPDH-F	TGCACCACCAACTGCTTAGC
GAPDH-R	GGCATGGACTGTGGTCATGAG
PPIA-F	CTCGAATAAGTTTGACTTGTGTTT
PPIA-R	CTAGGCATGGGAGGGAACA
PPIB-F	GCATGAAGGTGCCCCCCTCTTCGCC
PPIB-R	GCCTCCTTGGCAATGGCAAAGG
PPIL3-F	AAGACTGAGAAATCACGTAGTCC
PPIL3-R	CAAGCAGAAGGATGATGCAATC
β-actin-F	CACCATTGGCAATGAGCGGTTC
β-actin-R	AGGTCTTTGCGGATGTCCACGT

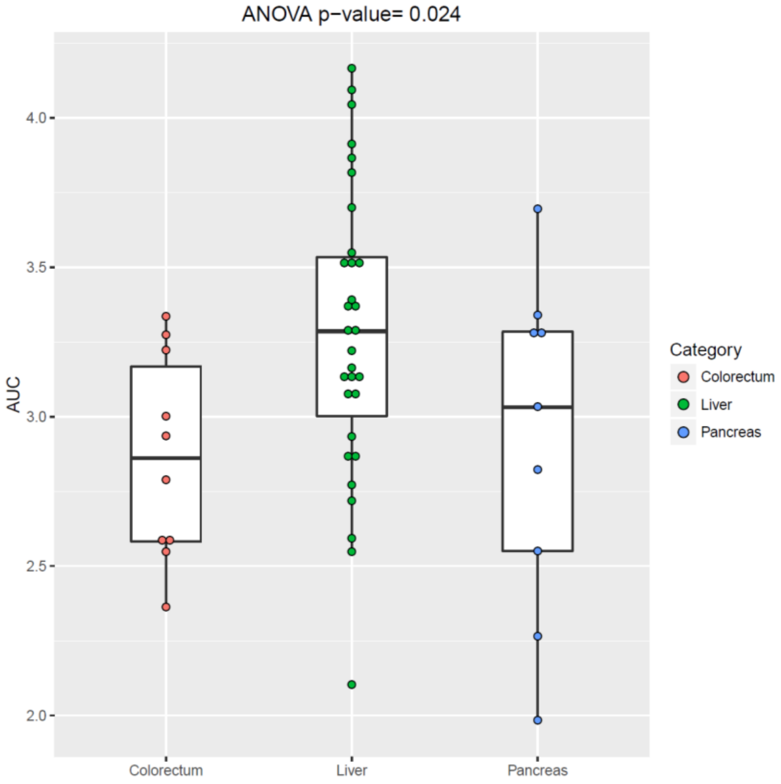

Figure S1. Boxplot indicating average NV651 AUC and statistical analysis between different tumor types.

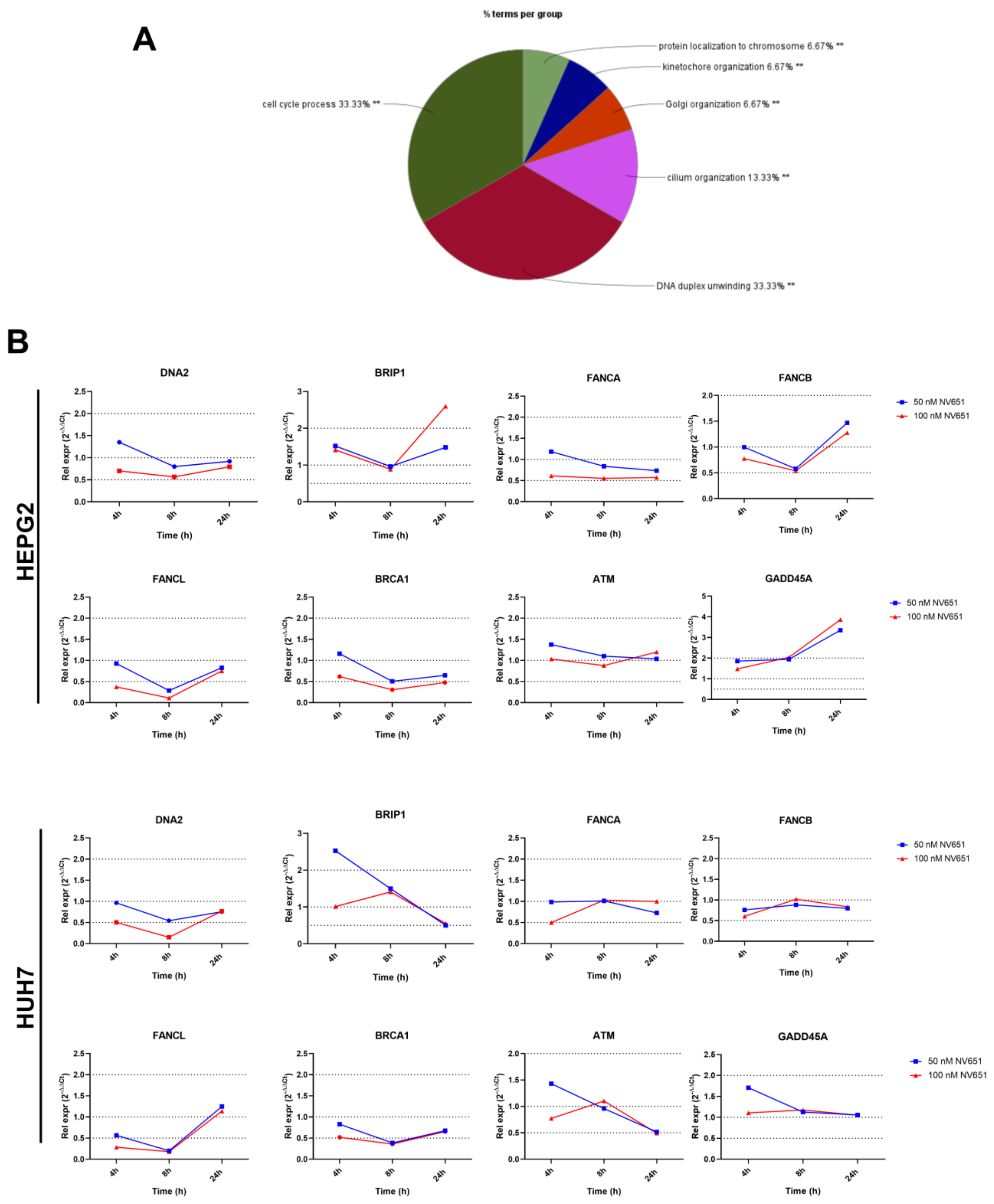

Figure S2. NV651 effect on gene expression in HCC cell lines. (A) Overlapped gene sets of GO-BP with a p-value lower than 0.05 were observed with ClueGO between NV651 versus control and NV651 vs CsA. (B) NV651 effect on expression in genes involved in DNA repair and cell cycle. HEPG2 and HUH7 cells were treated with 0, 50 or 100 nM of NV651 for 4, 8 and 72 hours. mRNA levels for each gene were evaluated by qPCR.

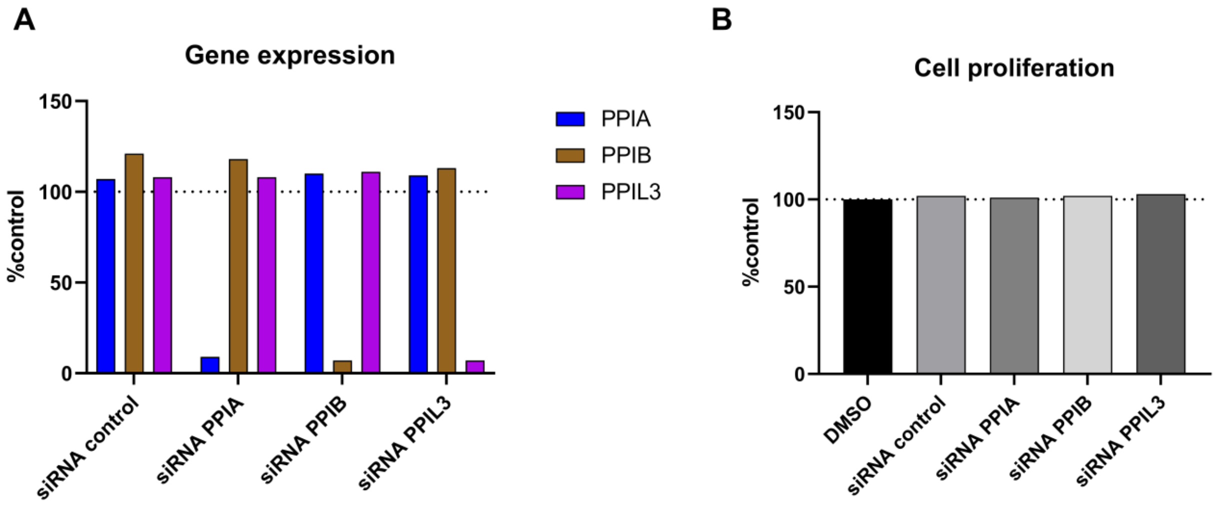

Figure S3. Gene silencing effect on cell proliferation (A) mRNA levels (% DMSO control) of PPIA, PPIB and PPIL-3 and normalized with β-actin. qPCR was performed after 24 hours of siRNA addition. (B) Cell proliferation was evaluated by total cell number count and calculated in relation to the DMSO control (% control).

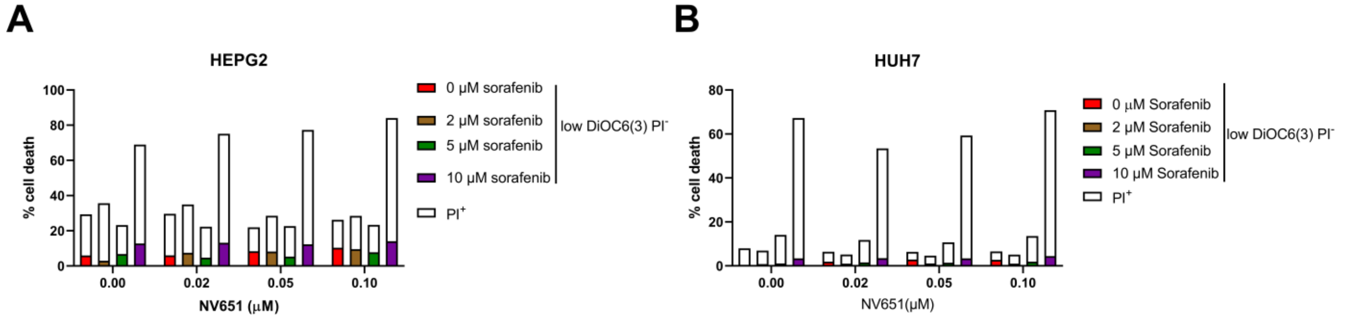

Figure S4. Sorafenib and NV651 effect on cell death. NV651 effect on the mitochondrial membrane potential (pre-apoptotic marker) and PI^+ cells (late apoptotic + necrotic marker) after 72 h of exposure to the combination treatment. Quantification of PI^+ cells and PI^- $_{low}$DiOC(6)3, equivalent to the low mitochondrial membrane potential in HEPG2 (A) and HUH7 (B).

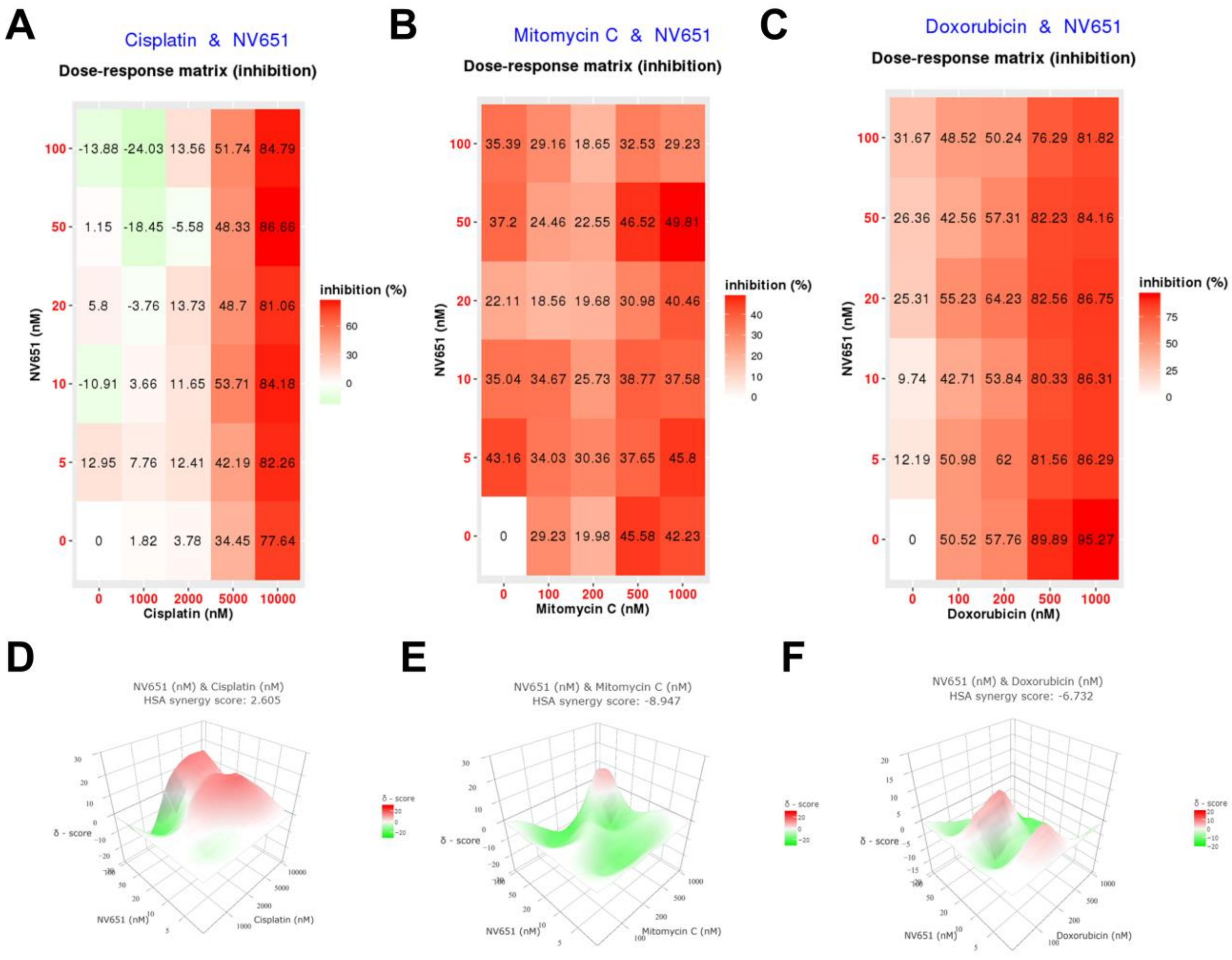

Figure S5. Synergistic effect with NV651 and HCC treatments in HUH7 cells. Effect on cell proliferation in HUH7 after 72 hours of exposure to the combination treatment between NV651 and cisplatin (A), Mitomycin (B) or Doxorubicin (C). Data in A-C are presented as % inhibition. D-F Synergy score calculated with highest single agent (HSA) from each data presented in A-C. n=1 biological replicate .

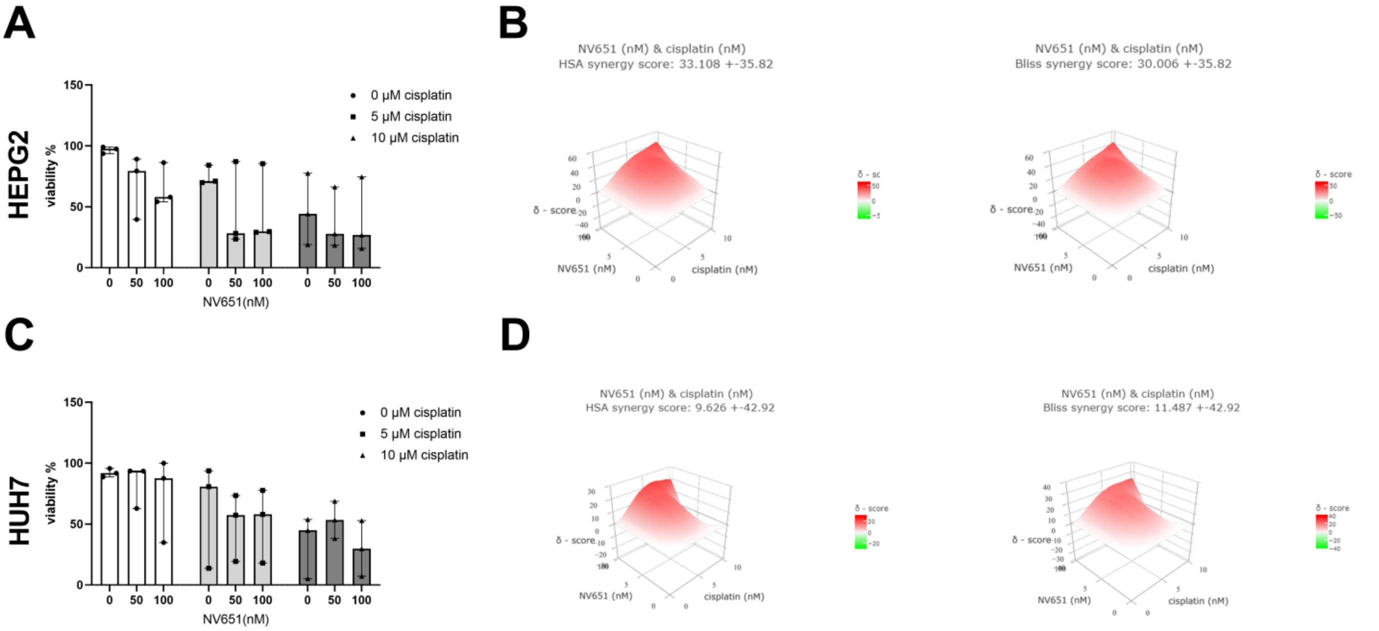

Figure S6. Effect on cell viability after 72 hours of treatment in HEPG2 (A) and HUH7 (C). Data are presented as median±95% CI of the percentage of viable cells negative for trypan blue. (B and D) Synergy score calculated with highest single agent (HSA) and Bliss from data presented in A and C. n=3 biological replicates.

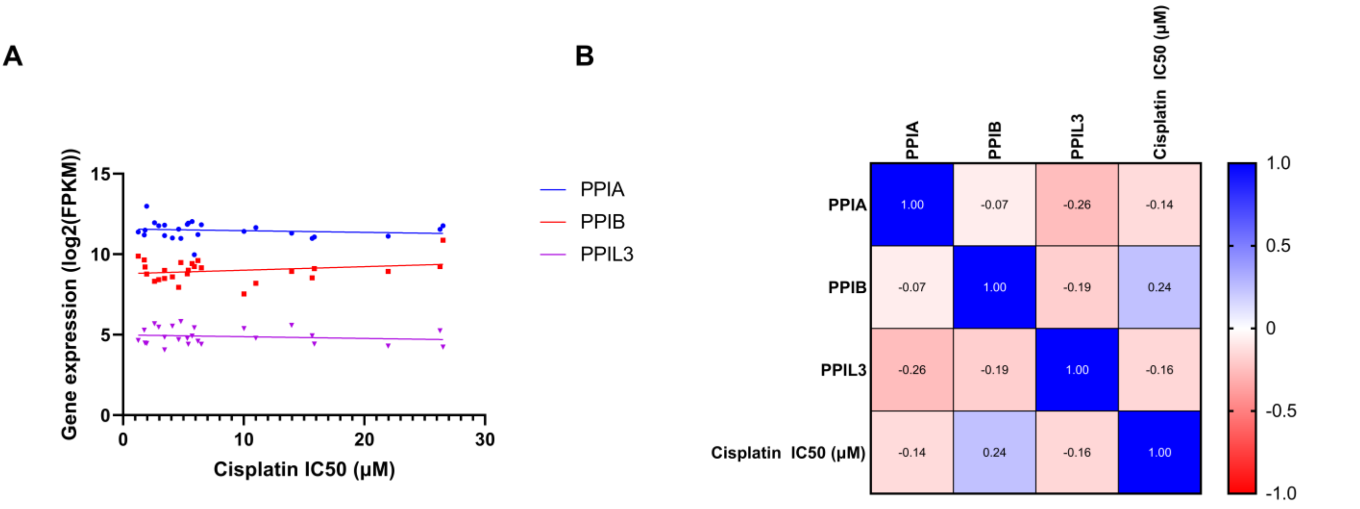

Figure S7. Correlation between cisplatin sensitivity and Gene expression of PPIA, PPIB and PPIL3 from Xenobase (Crownbio) (A) Median IC50 of cisplatin and gene expression of PPIA, PPIB and PPIL3 of available cancer cell lines originated from the liver are represented by each data point. (B) Pearson r/ correlation coefficient between cyclophilin expression and cisplatin sensitivity indicated by IC50. Colour range indicates the R value .
